# A Herd Investigation Tool in Support of the Irish Bovine Viral Diarrhoea Eradication Programme

**DOI:** 10.3389/fvets.2021.694774

**Published:** 2021-08-17

**Authors:** Maria P. Guelbenzu-Gonzalo, Jose-Maria Lozano, Padraig O'Sullivan, Elizabeth A. Lane, David A. Graham

**Affiliations:** ^1^Animal Health Ireland, Carrick-On-Shannon, Ireland; ^2^Central Veterinary Research Laboratory, Department of Agriculture, Food and the Marine, Backweston Laboratory Complex, Celbridge, Ireland; ^3^Irish Cattle Breeding Federation, Bandon, Ireland; ^4^Animal Health Division, Department of Agriculture, Food and the Marine, Dublin, Ireland; ^5^Centre for Veterinary Epidemiology and Risk Analysis, School of Veterinary Medicine, University College Dublin, Dublin, Ireland

**Keywords:** bovine viral diarrhoea virus, investigation, biosecurity, control programme, eradication

## Abstract

Bovine viral diarrhoea (BVD) is an important endemic disease of cattle. In Ireland, an industry-led compulsory eradication programme began in January 2013. The main elements of this programme are the identification and elimination of persistently infected (PI) calves by testing all new-borns, the implementation of biosecurity to prevent re-introduction of disease and continuous surveillance. In 2016, a standardised framework was developed to investigate herds with positive results. This is delivered by trained private veterinary practitioners (PVP). The investigation's aims are 3-fold: firstly, to identify plausible sources of infection; secondly, to ensure that no virus-positive animals remain on farm by resolving the BVD status of all animals in the herd; and thirdly, agreeing up to three biosecurity measures with the herd owner to prevent the re-introduction of the virus. Each investigation follows a common approach comprising four steps based on information from the programme database and collected on-farm: firstly, identifying the time period when each virus-positive calf was exposed *in utero* (window of susceptibility, taken as 30–120 days of gestation); secondly, determining the location of the dam of each positive calf during this period; thirdly, to investigate potential sources of exposure, either within the herd or external to it; and finally, based on the findings, the PVP and herdowner agree to implement up to three biosecurity measures to minimise the risk of reintroduction. Between 2016 and 2020, 4,105 investigations were completed. The biosecurity recommendations issued more frequently related to the risks of introduction of virus associated with contact with neighbouring cattle at pasture, personnel (including the farmer), the purchase of cattle and vaccination. Although each investigation generates farm-specific outcomes and advice, the aggregated results also provide an insight into the most commonly identified transmission pathways for these herds which inform overall programme communications on biosecurity. The most widely identified plausible sources of infection over these years included retained BVD-positive animals, Trojan births, contact at boundaries and indirect contact through herd owner and other personnel in the absence of appropriate hygiene measures. While generated in the context of BVD herd investigations, the findings also provide an insight into biosecurity practises more generally on Irish farms.

## Introduction

Bovine viral diarrhoea (BVD), caused by the BVD virus (BVDV), is endemic in many parts of the world (1). Infections with BVDV cause significant economic losses which result from its reproductive effects and exacerbation of concurrent bacterial or viral infections (2). The virus is spread mainly by persistently infected (PI) animals, established following infection *in utero* between 30 and 120 days of gestation (3), which continuously shed large amounts of virus after birth. These PI animals are the most common source of infection for other animals, as the virus is excreted in a wide range of bodily fluids including nasal discharge, urine, faeces, milk, semen, saliva, tears and foetal fluids (4). Transiently infected (TI) animals are considered to be poorer transmitters of the infection (5, 6). The most effective means of transmission is by nose-to-nose contact, although venereal transmission and indirect transmission through fomites and people have also been reported (7, 8). Naïve pregnant dams that experience a transient infection and are consequently carrying a PI foetus and which are then introduced to another herd are called “Trojan” dams. While the dam develops an immune response and appears healthy, they are important from an epidemiological perspective since they will deliver a PI calf in the herd to which they have been introduced (9).

Several BVD control/eradication programmes are in place or have been completed in Europe (10, 11). Their organisation differs between countries and regions due to variation in factors such as initial prevalence, structure of the cattle industry (density, extent of animal movements, etc.) and willingness of the government to support them financially or through legislation. A systematic approach, comprising identification and removal of PI animals, the application of appropriate biosecurity measures (potentially including vaccination) and ongoing monitoring to ensure that uninfected herds remained free from infection (12), is now widely adopted.

An industry led compulsory BVD eradication programme began in Ireland in January 2013 after 1 year of voluntary participation. The programme is explained in detail elsewhere (13, 14). Key elements include the identification and removal of persistently infected (PI) calves by testing all new-borns, the implementation of biosecurity to prevent re-introduction of disease and ongoing surveillance. Through legislation, only animals that have a negative BVD status can move out of farms, thus preventing a key means of introduction of infection into naïve herds (15). Therefore, the main risks of introduction to farms originate from introduction of Trojan dams, transiently infected animals or animals that tested negative for virus but are actually PI (apparent false negatives), and direct or indirect contact with infected animals in other herds.

In 2016, a standardised framework supported by a range of tools on the programme database was developed to investigate herds where one or more calves returned a virus-positive result. This Targeted Advisory Service on Animal Health, funded through the Rural Development Programme is delivered by trained private veterinary practitioners (PVP). The investigations' aims are 3-fold: firstly, to identify plausible sources of infection for the birth of PI calves; secondly, to ensure that no virus-positive animals remain on farm by resolving the BVD status of all animals in the herd; and thirdly, agreeing up to three biosecurity measures with the herd owner to prevent re-introduction of the virus.

Each investigation follows a common approach comprising three steps based on information from the programme database and collected on-farm. Firstly, identifying the time period when each calf was exposed *in utero* (window of susceptibility, taken as 30–120 days of gestation); secondly, determining the location of the dam of each positive calf during this period; thirdly, taking the outcomes of two previous steps into account, to investigate potential sources of exposure, either within the herd or external to it. Based on the findings, the PVP and herdowner agree to implement up to three biosecurity measures to minimise the risk of reintroduction.

The aims of this report are 2-fold, namely, to describe the herd investigation process and to summarise key findings from those completed between 2016 and 2020.

## Materials and Methods

### Programme Database

**Figure 1:**
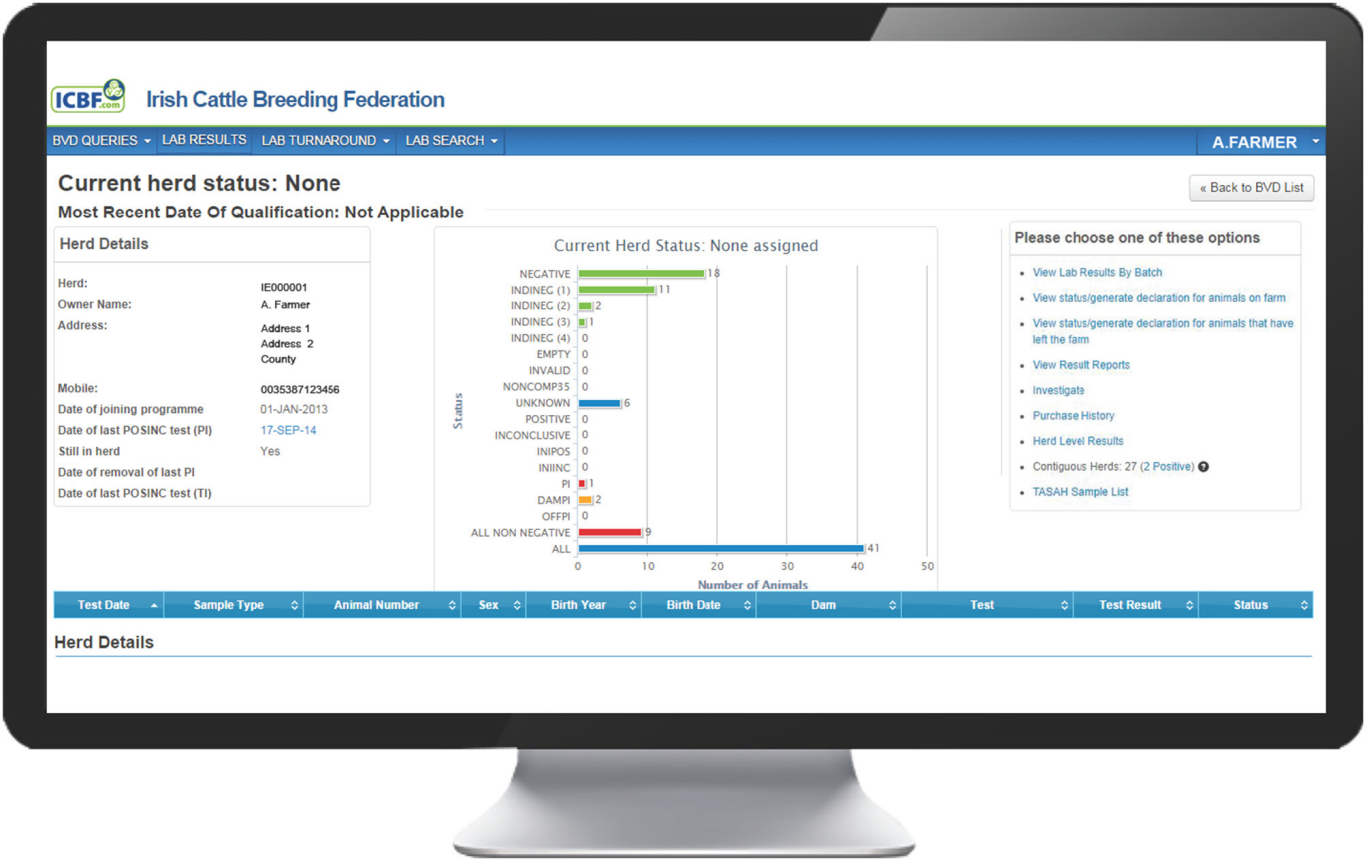
Landing page on the BVD herd-level dashboard provided by ICBF, summarising the current status of all animals in the herd and providing a series of additional options.

The programme database provided by the Irish Cattle Breeding Federation (ICBF^1^) manages key elements of the programme. Results from all testing laboratories are received by the database and are used to assign one of 13 possible mutually exclusive statuses to each individual animal (Table 1), taking into account both its own test results and those of its offspring and dam [e.g., assigning an indirect negative status (INDINEG) to a dam on the basis of a direct negative result for a calf]. A herd-specific dashboard is available to each herd owner on the database, which graphically presents the status of all animals currently in the herd (Figure 1), along with key summary statistics and a range of additional options. Full details of each animal, including age, sex and test history are available, alongside all programme communications, information on contiguous herds and details of all animals, and their dams, that have had a positive or inconclusive virus test result by either antigen ELISA or RT-PCR (via the “Investigate” option). Test results are classified as positive, inconclusive or negative based on the manufacturers' guidelines for the respective tests.

**Table 1:** Summary of the 13 possible statuses assigned to each animal in the programme database in relation to its BVD status, and the interpretation and action recommended with each one.

**Status**	**Interpretation**	**Action**
DAMPI	Dam of an animal with a current positive (or inconclusive) result	Test to clarify dam status
EMPTY	No tissue in submitted sample (unsuitable for testing)	Re-test required. Tissue or blood
INCONCLUSIVE	Current inconclusive result on database where initial result was not positive/inconclusive (e.g., initial empty result)	Isolate; option to re-test after 3–4 weeks to confirm PI
INDINEG 1, 2, 3, N	Dam that has produced 1, 2, 3, N negative calves (not PI)	–
INIINC	Initial test result is inconclusive, no re-test result	Isolate; option to re-test after 3–4 weeks to confirm PI. Isolate and remove as soon as possible
INIPOS	Initial test result is positive, no re-test result	Isolate; option to re-test after 3–4 weeks to confirm PI. Consider removal without retest
INVALID	Result not valid	Re-test required. Tissue or blood
NEGATIVE	Tested negative (most recent)	–
NONCOMP35	Animal without any test result 35 days after date of birth Re-test required. Tissue or blood	Test required by legislation
OFFPI	Untested offspring of a dam with a current positive (or inconclusive) result	Isolate and remove as soon as possible
PI	Initial and confirmatory positive (or inconclusive) result	Isolate and remove as soon as possible (<3 weeks of first test)
POSITIVE	Current positive result on database where initial result was not positive/inconclusive (e.g., initial empty result)	Isolate; option to re-test after 3–4 weeks to confirm PI. Consider removal without retest
UNKNOWN	(1) Born before 1st January 2013 and has not been tested and has not calved OR (2) a calf that has been born <35 days ago without any test result	(1) Test to clarify status (result required for Negative herd status if it remains in herd) (2) Test required by legislation

Each veterinary practice also has access to herd-level dashboards through a BVD practice dashboard, which provides a live listing of the status of all herds to which the practice has been granted access. These dashboards are also available to Animal Health Ireland^2^ (AHI) for programme management. Further details of the database functionality are described elsewhere (13, 14).

### Targeted Advisory Service on Animal Health

The Targeted Advisory Service on Animal Health (TASAH) is funded through the Rural Development Programme (2014–2020), co-funded by the Department of Agriculture, Food and the Marine (DAFM) and the European Commission, and is designed to provide farm-specific advice, provided by trained PVPs, on a range of diseases including BVD. The service is delivered through Animal Health Ireland, following successful participation in a DAFM-issued tendering process. This involves the training of PVPs, with this covering the epidemiology of the disease, the investigation protocol and the use of the programme database to support the investigation.

In addition, AHI oversees the co-ordination of the service. Each day, the programme database issues a list of herds for which a positive or inconclusive result has been received to a BVD Help Desk, staffed by DAFM personnel. Using a standard script, the Help Desk contacts the herd owner, ensuring that they are aware of the result (which is also issued directly from the database by SMS and letter), informing them of the requirement for an investigation and recording the identity of the trained PVP nominated by the herd owner to carry out their herd investigation and any associated sampling.

Details of the nominated PVP are in turn logged in AHI's Customer Relationship Management system (CRM, Microsoft Dynamics 365) which issues an email to the PVP providing details of the requested investigation. Trained PVPs have access to this CRM via a Service Provider Portal^2^ where they can manage their own investigations and access all the relevant paperwork, leaflets and training materials through the BVD module.

### Herd Investigation

Each investigation follows a standardised approach comprising four steps and based on information from the programme database and collected on-farm.

Firstly, the time period when each calf was exposed *in utero* [window of susceptibility (WOS), taken as 30–120 days of gestation] is identified. Secondly, the location of the dam of each positive calf during this WOS is investigated. Thirdly, taking the outcomes of two previous steps into account, potential sources of exposure, either within the herd or external to it, are investigated. As part of this step, on-farm sampling may be carried out to either determine the status of animals for which this is currently unknown or to minimise the possibility that any animals with a false-negative result are present. Fourthly, based on the findings of the investigation, the PVP and herdowner agree to implement up to three biosecurity measures to minimise the risk of reintroduction. Further detail of these steps is provided below.

A detailed protocol for this process is provided to trained PVPs, along with a herd investigation worksheet (both available from the corresponding author on request). The herd investigation worksheet is primarily designed to provide a structured framework for the conduct of the herd investigation following the birth of a BVD+ calf (i.e., a calf that has had an initial virus positive or inconclusive result and either has been removed without a retest or has been confirmed as PI on a retest). This worksheet is essentially a structured questionnaire, presented as a fillable pdf form, which ensures that all relevant data are collected and guides the investigating PVP through each step of the process. At appropriate points, it directs the PVP to the section of the programme database where data relevant to the particular step is located. Within the worksheet, mandatory questions to complete are highlighted. Where relevant, answers that indicate an increasing biosecurity risk are marked in red, while those associated with a lowering of risk are in green.

Additional supporting documents including a standard operating procedure (SOP), guides (including on vaccination, measures to minimise the risk of Trojan introduction and bioexclusion) and access to the training materials are available to the PVP via the Service Provider Portal.

#### Determining the Period of Exposure in utero

The first step in the investigation is to determine the time when the dam of the BVD+ calf was exposed to BVDV. Assuming that the dam is not herself persistently infected with BVDV, each BVD+ calf has been born as a result of exposure of their dam during the WOS in early pregnancy, typically between 30 and 120 days of gestation (3).

Selecting Investigate from the options available on the herd dashboard (Figure 1), opens a screen showing a range of information on each BVD+ calf and its dam.

**Figure 2:**
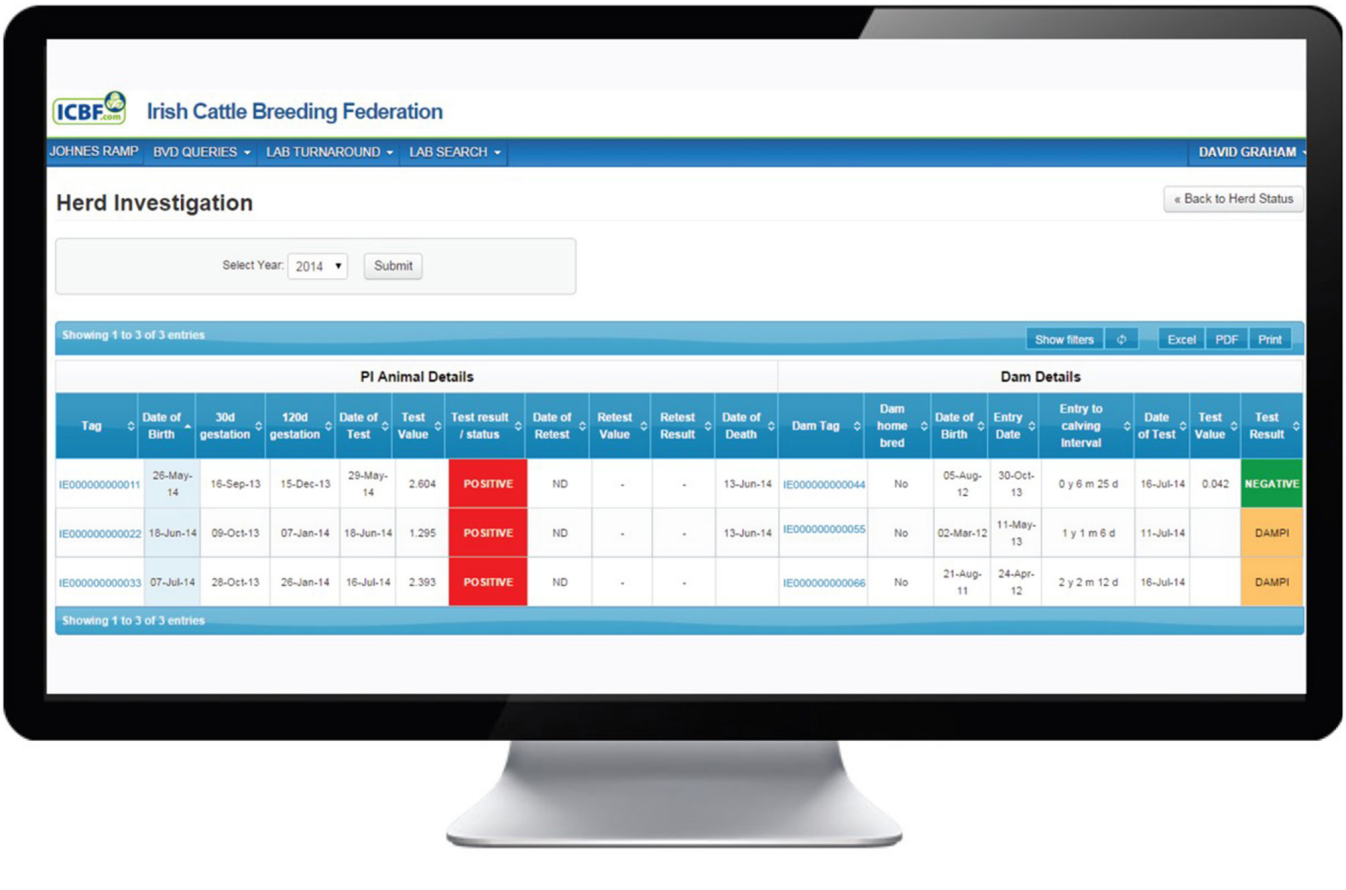
Investigation screen on the BVD herd-level dashboard provided by ICBF, providing details of each animal with a positive or inconclusive result in the herd, including date of commencement and conclusion of the window of susceptibility, and associated dam details.

The Investigate function may be used to view data for a particular year or for all years (Figure 2). Every animal with a positive or inconclusive result is listed. Based on the recorded birth date for each calf and a 282-day gestation, the dates of opening (30 days) and closing (120 days) of the WOS are shown. However, investigating PVPs are advised that while these are the generally accepted limits, they should not be treated as absolute time boundaries. Additional fields provide the date and results of the initial and any subsequent tests and, where relevant, the date of removal from the herd. In addition, for the dam of each listed animal, its date of birth, if it is homebred or not, its date of entry to the herd, the interval from entry to calving (i.e., date of birth of the test positive/inconclusive calf) and its test history are provided. This information can be used to identify the cohort (heifer, cow) to which the dam belongs and to explore the possibility of births to Trojan dams (either to non-home bred animals introduced to the herd or homebred heifer returning from being contract reared in another herd). Furthermore, this screen gives access to a family tree function showing the ancestors or descendants of a given animal by sex, date of birth, date of death and BVD status.

Where more than one BVD+ animal has been born, these can be sorted alphanumerically in each column, e.g., by date of birth or date of removal. Where there have been multiple positive calves born with overlapping windows of susceptibility, the source of infection could potentially have been continuously present from the date that the first dam entered the WOS to the date that the last dam left the window. This would be the expected situation where the source of infection is internal to the herd, e.g., the presence of an unidentified BVD+ animal in the herd.

Alternatively, the birth of multiple positive calves could also occur if the source of infection was present for a shorter period of time while all dams were within the window of susceptibility. This could arise where infection originated from a “point source” as a result of a one-off event, e.g., an animal breaking in or boundary contact.

#### Determining the Location of Exposure

In discussion with the herd owner, the investigating PVP will determine the location of the dam(s) during the WOS identified in the previous step. In the case of Trojan dams, this would have happened outside the herd as the dams were pregnant when introduced. Where the animal was <120 days in calf when introduced, it is possible that the foetus became infected after introduction; therefore, this animal will be a considered a possible Trojan dam. Where the animal was more than 120 days in calf, it is highly likely that it was carrying a BVD+ calf when introduced; hence, this animal will be considered a definite Trojan dam.

The age of the investigated dam(s) will indicate the particular management group or groups that were exposed. If all these animals were managed as a single group, this suggests the contact of only this group with a source of virus (e.g., a batch of heifers on an out-farm). Where dams have a range of birth dates, this suggests the exposure of the adult herd and/or multiple management groups to a common source of virus.

Other important questions include whether during this period, the dams were on the home farm or an out-farm, housed or at pasture, grazing contiguous to farm boundaries or outside the herd for part or all of this period (e.g., for contract-rearing of heifers).

#### Investigating Potential Sources of Exposure

**Figure 3:**
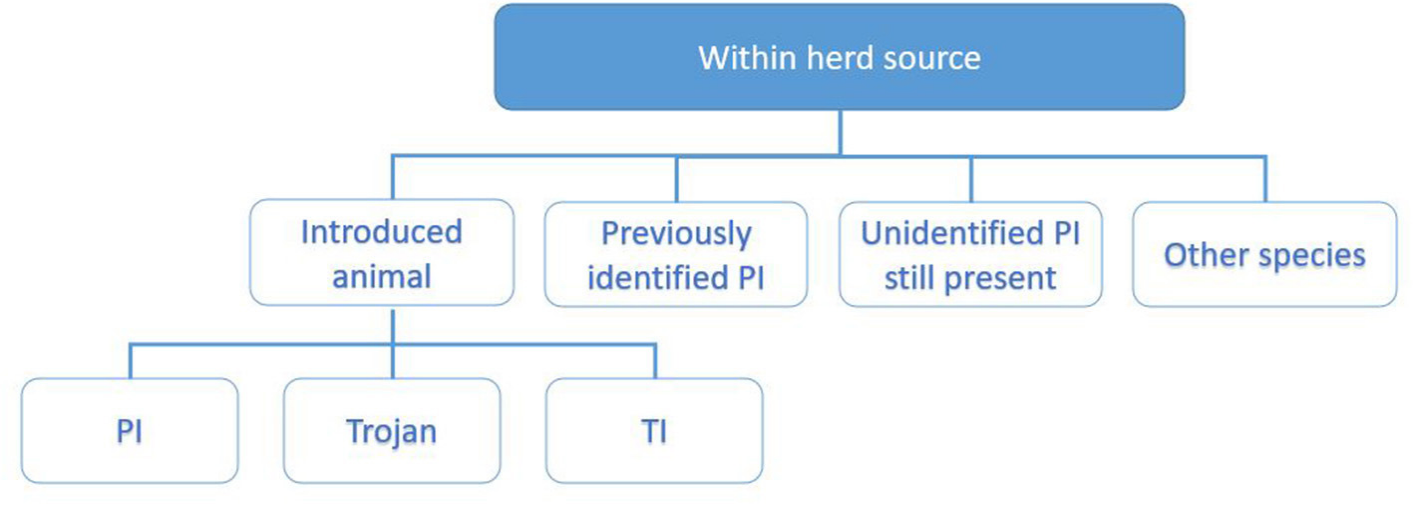
Categorisation of potential within-herd sources of infection explored during the investigation. These include introduced animals (9, 15, 16), previously identified PI (17), unidentified PI still present (14), and other species (18, 19).

**Figure 4:**
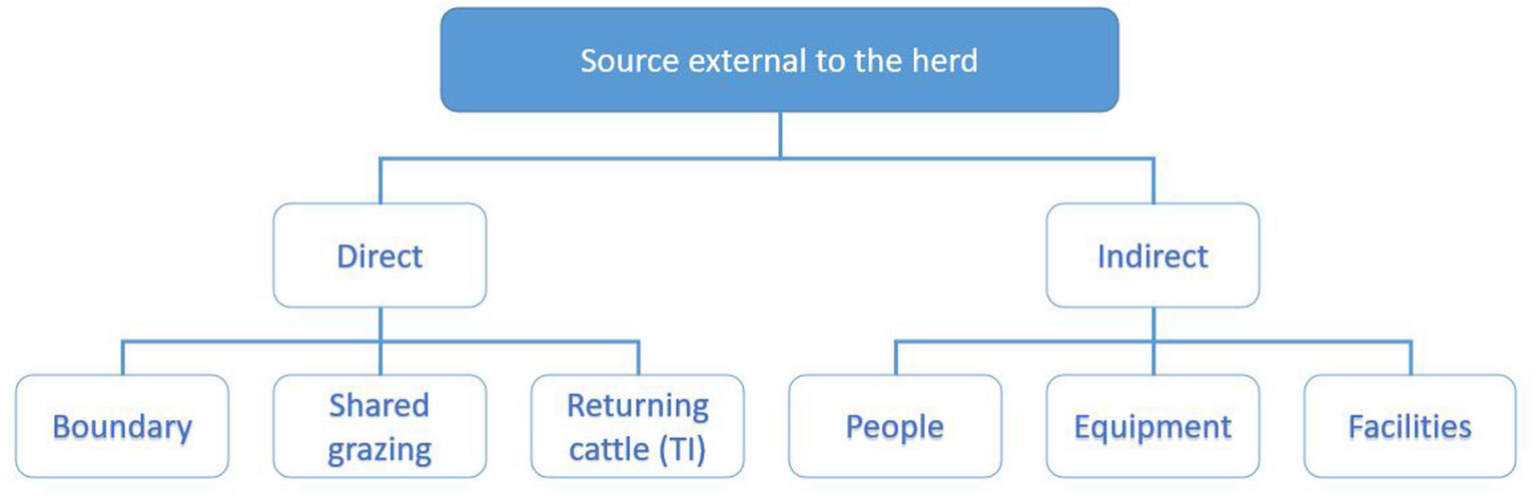
Categorisation of potential sources of infection external to the herd explored during the investigation. Direct sources of infection include boundaries (20), shared grazing (11) and returning cattle (15). Indirect sources include people, equipment and facilities (7, 8).

The investigating PVP will collate these data by interviewing the herd owner following the investigation worksheet, supported with data from the programme database and the associated sampling results. For the purpose of working through the potential sources of infection, these are divided into sources within the herd (Figure 3) and outside the herd (Figure 4) and each investigated in turn as described below.

##### Within Herd Source

*Presence of Unidentified BVD + Cattle in the Herd*. One of the main objectives of the herd investigation is to ensure that the herd is left free from BVDV. The programme database allows the rapid identification of animals in the herd without a negative BVD status, being summarised as “all non-negative” on the dashboard graphic (Figure 1). A list of these may be generated and exported in Excel or pdf format to convert this to a saveable file in the chosen format or “print” to generate an immediate hard copy and to facilitate identification and sampling. All of these animals should be blood-sampled and tested for the BVD virus either by antigen ELISA or RT-PCR as part of the investigation. From 2016 to 2018, the sampling associated with herd investigations comprised the re-test of animal(s) with virus-positive or inconclusive results, their dams for BVD virus and antibodies, and animals of non-negative BVD status, i.e., those with the following statuses: EMPTY, INVALID, NONCOMP35, UNKNOWN, INTRODUCED35, DAMPI and OFFPI (Table 1).

In 2019 and 2020, following the identification of a small number of animals with apparent false-negative (AFN) results over the course of the programme, an additional requirement was introduced to test animals that could potentially have a false-negative status recorded on the database. This additional sampling included animals that have had a single negative BVD status (assigned directly or indirectly) and that were present in the herd during the relevant WOS. Animals with a single direct negative test which had also produced one or more calves that have also tested negative were excluded from this sampling. Animals were blood-sampled and tested by antigen ELISA or RT-PCR. Additional functionality was developed on the herd dashboard to generate a full listing of animals to be sampled by selecting the “TASAH Sample List” option (Figure 1). In dairy herds, in addition to the blood sampling, a bulk tank milk sample was taken to be tested by RT-PCR for the presence of the BVD virus. This service is provided essentially at no cost to the herd owner. The epidemiological investigation itself is funded through the Rural Development Programme (2014–2020), while the additional sampling and testing is funded by DAFM or provided without charge through the National Reference Laboratory for BVD.

*Contact With a Known BVD + Animal*. This could occur where a BVD+ animal had been born previously in the herd, overlapping with the WOS of the case being investigated. The retention of virus-positive calves born in the previous calving season has been shown to increase the probability of finding a virus-positive animal in a herd (21). The Investigate screen indicates firstly if there were previous BVD+ calves born in the herd, and if so, a review of the relevant dates of birth and removal indicates if overlap occurred.

*Introduced Animals*. Introduction of animals has been highlighted before as one of the main factors associated with the presence of BVDV (9, 20, 22). While the animals themselves have come from outside the herd, at the time of the investigation they are in the herd and therefore included as part of the investigation of within-herd sources. Potentially, these introductions could be in the form of a PI, a Trojan dam or a transiently infected animal.

As already described, the possibility of Trojan births can be explored through the dam details on the Investigate screen. If the BVD+ calf was born to an introduced animal, the “entry to calving interval” should be checked. If this is <282 days, it is possible that the dam was a Trojan. If the dam was introduced <162 days from calving (i.e., when more than 120 days in calf), the WOS would have closed before the dam joined the herd and she was regarded as a definite Trojan. Where the interval is >162 days, the dam is considered a potential Trojan as the possibility that infection occurred after introduction cannot be excluded.

**Figure 5:**
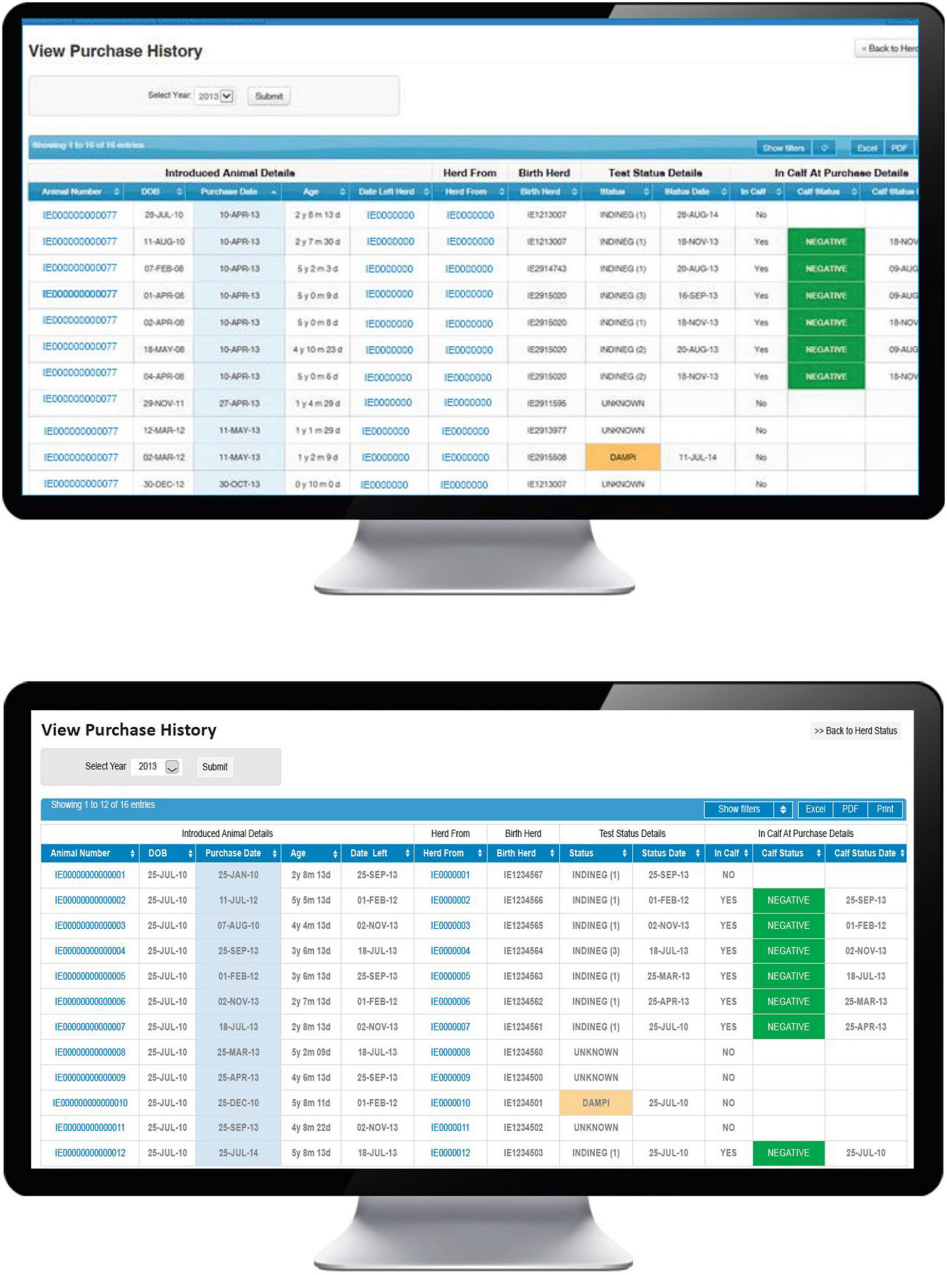
Purchase history screen on the BVD herd-level dashboard provided by ICBF, listing details of introduced animals including purchase date, pregnancy status at purchase, and their current test status.

A number of steps are necessary for this pathway to result in a PI birth, beginning with the introduction of animals immediately prior to or during the relevant WOS, their being TI at the time of introduction and, thereafter, the possibility of transmission of virus to the relevant dam. In the first instance, the PVP will use the “Purchase history” option on the BVD dashboard (Figure 5) to view a full listing of all introduced animals. Sorting this information by purchase date allows the PVP to determine if any animals were introduced during the WOS and to review further information on any such animal, including its date of birth, date of introduction, current age, date of departure from the current herd (where relevant), identity of its birth herd, its most recent test date (by antigen ELISA or RT-PCR) and status and if it was in calf at purchase (based on first recorded calving date after introduction) and where relevant, the test status of this calf (also determined by either antigen ELISA or RT-PCR). Where home-born animals have left the herd under investigation and subsequently returned (e.g., from a contract rearer or associated herd) the number of the herd under investigation will be shown as the birth herd.

When relevant introductions are identified, the PVP will gather further information to determine if this is a plausible source of infection, including whether the introduced animals moved directly from the farm of origin or they had the opportunity to mix with cattle from other herds, e.g., at markets or during transport; if a quarantine policy is in place for added animals (also its duration and whether it was actually applied to the introduced animals); if the introduced animals were tested for BVD virus and/or antibody; and how long after introduction did the added animals first have contact with the dam(s) that went on to produce the BVD+ animal(s).

*Other Species*. BVD virus is predominantly associated with cattle but it can infect other ruminant species (sheep, goats, llamas, alpacas) and pigs (23, 24). Sheep may also be infected with Border disease virus (BDV), a pestivirus related to BVDV and which has occasionally been detected in cattle in other countries, although not in Ireland to date. BVDV and BDV can be found in sheep as well as in cattle and both viruses can be transmitted either way (sheep to cattle or cattle to sheep) (18, 19). The PVP will therefore ask a series of questions to determine if small ruminants are present on the farm, and if so, if they have contact through co-grazing, shared housing or only indirectly. If sheep are suspected as a source of infection, the investigating PVP is advised to consider carrying out serological screening for evidence of infection on a proportion of the flock.

##### Source External to the Herd.

The investigation considers six transmission pathways through which virus may be introduced directly or indirectly from sources external to the herd (Figure 4).

*Direct Contact*. Direct nose-to-nose contact with a PI animal is considered to be the most efficient route for the transmission of the virus (25, 26). Taking the time period of the WOS identified in step 1, and the location(s) where exposure may have occurred identified in step 2, three potential sources of direct contact are investigated: at boundaries, on shared grazing and through returning cattle.

A sequence of questions explores the potential for transboundary transmission, including: if the dam was grazing at a boundary during the WOS; the presence of neighbouring cattle on the other side of the boundary at that time; the quality of the boundary [sufficient to prevent nose to nose contact (3-m gap) or the break in (or out) of cattle] and any known instances of cattle mixing following boundary breaches.

The investigating PVP has access to information on BVD+ births in neighbouring herds through the “Contiguous Herds” option on the BVD dashboard (Figure 1). This shows the total number of contiguous herds, the number of these that have had animals with INIPOSINC results since April 1, 2016 (commencement of the TASAH investigations) and the dates of birth and death of each of these animals. This information, along with that already gathered, helps to determine whether transmission across a boundary is a plausible source of infection or not, being excluded in the absence of positive contiguous herds. In addition, knowledge of the status of contiguous herds also assists the PVP when considering indirect transmissions pathways.

Use of shared grazing is explored directly with the herd owner, who is also asked about the possibility of cattle leaving the herd and returning subsequently, e.g., from shows, unsold from sale or after contract rearing or leasing out [supported by analysis of information on the purchase (strictly, introduction) screen]. Where relevant, they are also asked whether those cattle had the opportunity to contact cattle of unknown status from other herds during this time; if they were quarantined prior to reintroduction to the main herd and how long after return they first had contact with the dams that produced BVD+ calves.

*Indirect Contact*. Although indirect transmission of BVDV is thought to be much less efficient, it has been demonstrated before (7, 27). Three indirect transmission pathways are investigated related to the movement or sharing of people, equipment and facilities.

In relation to people, the herdowner's own possible role is investigated first, including their contact, directly or indirectly with cattle in other herds and, where relevant, the level of biosecurity/hygiene applied to manage this risk (changing or disinfecting boots and clothing, washing hands).

Next, the PVP explores the number and type of visitors to the farm during the WOS, including farm employees and relief workers, knackery staff, AI technicians, hoof trimmers, weighing technicians, PVPs, advisors, nutritionists, etc. Where relevant, this was explored further in terms of their actual contact with the cattle in general and the dams that produced PI calves in particular; the frequency of this contact and the level of biosecurity/hygiene was applied to/demonstrated by these visitors on arrival (and departure); for example, whether boots and clothing were provided by the herd owner for on-farm use; if routine disinfection of visitors' boots, clothing and equipment was taking place; or if hand washing was practised. Taking all of this information into consideration, the PVP assigns each visitor a risk ranking from very low to high.

Herd owners are also asked if, during the WOS, they had used items of borrowed equipment, either small (e.g., nose tongs, calving aids, drenching guns, dehorning, or foot paring equipment) or large (e.g., trailers used to move cattle), or had shared facilities with other herds, particularly housing, yards or crushes. Where relevant, additional questions determined if these had been cleaned and disinfected before and after use.

### Identify Plausible Sources, Review Biosecurity, and Make Recommendations

The investigating PVP also captures information on the herd's BVD vaccination status, including the product used and for how long a vaccination regimen has been in place. Then, having completed the investigation and review of BVD-related biosecurity on farm, the PVP formulates and agrees on up to three measures to improve herd biosecurity with the herd owner.

In addition to these measures, the PVP will reinforce advice to minimise the risk of the sale of Trojan dams from these herds. Specifically, herd owners are advised that they should not sell animals that were pregnant at the time of removal of the last BVD+ animal unless they are antibody negative within 2 weeks of sale.

When the investigation for a herd has been completed, the PVP enters key findings (including all responses to the mandatory questions in the worksheet), details of the agreed biosecurity measures, plausible sources identified and details of the numbers and type of samples submitted on the CRM. This generates a further email to the PVP, providing a summary of the biosecurity findings with an instruction to ensure they are provided to the herd owner.

### Data Management

Findings are recorded by the investigating PVP in Animal Health Ireland's CRM system through the BVD module of the Service Provider Portal, accessible through AHI's website. The data are extracted and analysed on a monthly basis by AHI. The monthly report includes the number of investigations requested and completed, the total number of positive herds and the number of samples collected for the year to date. Results are reported to the BVD Technical Working Group and/or BVD Implementation Group as necessary and are used to inform common biosecurity messages.

### Data Analysis

Questionnaire answers and findings recorded by PVPs for 4,105 investigations completed between 2016 and 2020 (including investigations received up to the January 12, 2021) were extracted from the CRM system of AHI. A descriptive analysis of the data was performed with Microsoft Excel®. Test results obtained from the sampling associated with the investigations were extracted from the ICBF database in Excel format.

## Results

Between 2016 and 2020, more than 540 PVPs were trained and 4,105 investigations were completed.

### Questionnaire Responses

The systematic BVD TASAH investigations were available for the first time in 2016, when nearly 50% of herds with a positive or inconclusive result went through the process (Table 2). As previously described (13), herds were considered to be dairy, beef or dual-purpose enterprises for the purpose of further analysis. A small number of herds which were not assigned to one of these three types were included with dual herds for presentation of results.

**Table 2:** Number (%) of positive herds overall and by herd type, number of BVD+ animals in these herds and the number (%) of these herds in which a BVD investigation was conducted each year and in total.

**Year**	**Positive herds**				**BVD+ animals**				Investigations conducted^*^			
	**Beef**	**Dairy**	**Dual**	**Total**	**Beef**	**Dairy**	**Dual**	**Total**	**Beef**	**Dairy**	**Dual**	**Total**
2016	1,203	790	217	2,210	1,898	1,515	399	3,812	619	380	103	1,102
	54.4%	41.6%	14.3%		49.8%	39.7%	10.5%		56.2%	34.5%	9.3%	
2017	669	505	127	1,301	1,100	1,046	251	2,397	669	560	151	1,380
	51.4%	45.9%	12.1%		45.9%	43.6%	10.5%		40.6%	40.6%	10.9%	
2018	377	286	74	737	587	597	140	1,324	375	314	72	761
	51.2%	48.7%	12.4%		44.3%	45.1%	10.6%		41.3%	41.3%	9.5%	
2019	255	190	52	497	421	475	92	988	269	205	54	528
	51.3%	45.1%	10.9%		42.6%	48.1%	9.3%		50.9%	38.8%	10.2%	
2020	188	136	35	359	339	311	70	720	158	146	30	334
	52.4%	40.1%	11.3%		47.1%	43.2%	9.7%		47.3%	43.7%	9.0%	
Total	2,692	1,907	505	5,104	4,345	3,944	952	9,241	2,090	1,605	410	4,105
	52.7%	37.4%	9.9%		47.0%	42.7%	10.3%		50.9%	39.1%	10.0%	

* *Note that some investigation conducted in a given year may have been undertaken as a result of positive findings in the previous year*.

The proportion of positive herds by herd type disclosed per year was similar every year. However, a higher percentage of positive beef herds underwent a BVD investigation in 2016 (56.2%) than in subsequent years (40.6% in 2017, 41.3% in 2018, etc.) (Table 2).

#### Introduction of Animals

Out of all investigations, 43.2% (1,771) reported having added animals to the herd immediately prior to or during the WOS (Table 3), with this being more common in beef (45.4%) than dairy herds (37.2%).

**Table 3:** Number (%) of positive responses to questions related to the introduction of animals, boundaries, visitors and personnel, and herd owner coming into contact with the cattle overall and by herd type.

	**Question**	**Answer**	**Beef**	**Dairy**	**Dual**	**Total**
Introduction of animals	Were any animals added to the herd immediately prior to or during the WOS?	Yes	948 45.4%	596 37.2%	227 55.4%	1,771 43.2%
	Did the introduced animals move directly from the farm(s) of origin or did they have the opportunity to mix with cattle from other herds (particularly those of unknown status)?	Mixed with other animals	642	242	157	1,041
			67.7%	40.7%	69.2%	58.8%
		Moved directly	306	353	70	729
			32.3%	59.3%	30.8%	41.2%
	Does the investigation herd have a quarantine policy for introduced animals	Yes	163	117	29	309
			17.2%	19.6%	12.8%	17.5%
	If YES, was it applied to the introduced animals?	Yes	148	95	26	269
			90.8%	81.2%	89.7%	87.1%
	Were the introduced animals tested for BVD virus?	Yes	61	45	7	113
			37.4%	38.5%	24.1%	36.6%
	Were the introduced animals tested for BVD antibody?	Yes	15	10	3	28
			9.2%	8.6%	10.3%	9.1%
Boundaries	Were the cattle grazing at a boundary at any time during this period?	Yes	1,628	1,339	327	3,294
			77.9%	83.5%	79.8%	80.3%
	If YES,					
	i) Were there cattle from neighbouring herds on the other side of the boundary at that time?	Yes	1,211 74.3%	1,021 76.2%	246 75.2%	2,478 75.2%
	ii) Did any of these neighbouring herds contain PIs at this time?	Yes	92 5.7%	89 6.7%	21 6.4%	202 6.1%
	iii) Was the quality of the boundary sufficient to prevent nose to nose contact (3M gap)?	Yes	711 43.7%	765 57.1%	168 51.4%	1,644 49.9%
	iv) Was the quality of the boundary sufficient to prevent break in (or out) of cattle?	Yes	915 56.2%	738 55.2%	184 56.3%	1,837 55.8%
	Are any break-ins or outs known to have occurred during this period?	Yes	239	235	47	521
			14.7%	17.6%	14.4%	15.8%
	Shared grazing: Do the cattle share grazing with other herds (e.g., commonage)?	Yes	50	8	7	65
			2.4%	0.5%	1.1%	1.6%
Visitors, personnel	Farm employees	Yes	539	724	164	1,427
			25.8%	45.1%	40.0%	34.8%
	Farm relief workers	Yes	125	342	38	505
			6.0%	21.3%	9.3%	12.3%
	Knackery staff	Yes	386	533	124	1,043
			18.5%	33.2%	30.2%	25.4%
	AI technicians	Yes	537	647	110	1,294
			25.7%	40.3%	26.8%	31.5%
	Hoof trimmers	Yes	310	621	111	1,042
			14.8%	38.7%	27.1%	25.4%
	Weighting technicians	Yes	19	14	7	40
			0.9%	0.9%	1.7%	1.0%
	Veterinary practitioners	Yes	1,514	1,338	324	3,176
			72.4%	83.4%	79.0%	77.4%
	Advisors	Yes	139	275	48	462
			6.7%	17.3%	11.7%	11.3%
	Nutritionists	Yes	29	99	26	154
			1.4%	6.2%	6.3%	3.8%
	Other	Yes	256	152	51	459
			12.3%	9.5%	12.4%	11.2%
	No One	Yes	336	134	51	521
			16.1%	8.4%	12.4%	12.7%
Herd owner	During the WOS did the herd owner come in contact with cattle in other herds?	Yes	1,267	891	279	2,437
			60.7%	55.6%	68.0%	59.4%
	During the WOS did the herd owner attend shows, sales?	Yes	1,286	872	290	2,448
			61.6%	54.4%	70.7%	59.7%
	If YES, level of biosecurity/hygiene applied before interaction with cattle in their own herd: i) Change boots and clothing	Yes	408	351	91	850
			26.7%	32.6%	27.7%	29.0%
	ii) Disinfect boots and clothing	Yes	704	624	161	1,489
			46.0%	57.9%	49.1%	50.7%
	iii) Wash hands	Yes	1,221	898	277	2,396
			80.0%	83.4%	77.2%	81.7%

In 41.2% (729) of cases, the animals moved directly into the herd, while in the remaining 58.8% (1,041) the introduced animals mixed with animals from other herds, potentially including those of unknown health status. Dairy herds that introduced animals most commonly moved these directly from the farm of origin (59.3%), which is less common for beef herds that introduced animals (32.3%).

Of all the herds that introduced animals, 17.5% (309) had a quarantine policy, being recorded for similar proportions of dairy and beef herds. Of these 309 herds with a quarantine policy, 87.1% (269) had actually applied it to the introduced animals, while 36.6% of the 309 herds (113) had tested the introduced animals for virus and 9.1% (28) for BVD antibodies.

In herds with no quarantine policy (1,354), the time period after which introduced animals first had direct contact with the dam(s) that went on to produce a BVD+ calf was <1 week in the majority of occasions (63.5%, 860), with only a minority (21.3%, 288) reporting a period of 4 weeks or more (Table 4). In herds with a quarantine policy (309), this period was at least 4 weeks in 50.5% of cases (156). However, in 15.9% (49) of these herds the interval was <1 week and, in 14.9% (46), between 1 and 2 weeks.

**Table 4:** Time after introduction that introduced animals first had direct contact with the dam(s) that went on to produce a BVD+ calf according to reported presence or absence of a herd quarantine policy.

**Time to contact**	**Quarantine policy**					
	**No**		**Yes**		**Total**	
<1 week	860	63.5%	49	15.9%	909	54.7%
1–2 weeks	115	8.5%	46	14.9%	161	9.7%
2–4 weeks	91	6.7%	58	18.8%	149	9.0%
More than 4 weeks	288	21.3%	156	50.5%	444	26.7%
Total	1,354		309		1,663	

#### Boundaries

In 80.3% of investigated herds (3,294), dams of BVD+ calves were grazing at a boundary during the WOS, with a similar frequency between herd types (Table 3). 75.2% of herd owners were aware that cattle from neighbouring herds were present on the other side of the boundary at that time. In 202 (6.1%) investigations, the neighbouring herd were reported as containing PIs at that time. 49.9% (1,644) reported a sufficient boundary quality to prevent nose to nose contact and 55.8% (1,837) to prevent the break in or out of cattle. Conversely, 521 investigations (15.8%) reported known break-ins or -outs happening during the WOS. Only 1.6% (65) of investigations across the study period reported shared grazing with other herds.

#### Visitors, Personnel, and Herd Owners

PVPs were the most commonly reported personnel type that had contact with cattle (77.4% of investigations) during the WOS, followed by farm employees (34.8%) (Table 3). Dairy herds in general reported higher contact with people other than the herd owner than beef herds. This included farm employees (45.1% dairy vs. 25.8% beef), farm relief workers (21.3 vs. 6.0%), knackery staff (33.2 vs. 18.5%), AI technicians (40.3 vs. 25.7%), advisors (17.3 vs. 6.7%) and nutritionists (6.2 vs. 1.4%). Only a minority of herds (12.7%) reported no personnel having contact with cattle during the WOS, with this being more common in beef (16.1%) than in dairy herds (8.4%); 60.7% of beef and 55.6% of dairy herd owners reported coming into contact with cattle from other herds during the WOS and, separately, 61.6% of beef and 54.4% of dairy herd owners attended shows or sales during this period. Most herd owners that came in contact with cattle in other herds during the WOS reported washing their hands before interacting with cattle in their own herd (81.7%); 59.7% reported disinfecting boots and clothing [more common in dairy (57.9%) than beef (46%) herds], while 29.0% reported changing boots and clothing, with similar proportions between beef and dairy. Of herd owners who reported disinfecting boots and clothing (1,489), 47% (699) also changed them before coming into contact with their own cattle; 94% (1,401) of those who disinfected boots and clothing and 95% (807) of those who changed them also washed their hands prior to interacting with cattle in their own herd.

#### Equipment and Facilities

Only 8% of herds borrowed and used small items of equipment during the WOS (Table 5). Of these, only a minority (29.9%) reported cleaning and disinfecting them before and after use. Large items of equipment were also borrowed and used infrequently (19.7%), but again, where this did happen, only a minority (26.6%) of herd owners reported their being cleaned and disinfected. Just 5.5% of herd owners reported sharing facilities with other herds, but again, only a minority (17.0%) cleaned and disinfected those facilities before and after use.

**Table 5:** Number (%) of positive responses to questions related to the borrowing of equipment and sharing of facilities overall and by herd type.

	**Beef**	**Dairy**	**Dual**	**Total**
Were any small items of equipment (e.g., nose tongs, calving aid) borrowed and used during the WOS?	177	119	40	336
	8.5%	7.4%	9.8%	8.2%
If YES, were these cleaned and disinfected before and after use?	51	40	9	100
	28.8%	33.9%	22.5%	29.9%
Were any large items of equipment (e.g., trailers) borrowed and used during the WOS?	415	291	101	807
	19.9%	18.1%	24.6%	19.7%
If YES, were these cleaned and disinfected before and after use?	129	100	31	260
	26.3%	27.3%	25.8%	26.6%
Do animals in the herd share facilities with other herds (particularly housing, yards, and crushes)?	122	75	30	227
	5.8%	4.7%	7.3%	5.5%
If YES, were these cleaned and disinfected before and after use?	22	9	7	38
	18.2%	12.0%	25.0%	17.0%

#### Other Species

Across all years, 27.8% of beef herds (581) and 5.5% of dairy herds (88) reported having sheep present on the farm. Only 1.5% of herds (63) reported having goats, 0.2% (7) alpacas and 0.2% (8), llamas on farm. Of all of those with small ruminants, 74.5 and 44.4% co-grazed with cattle in beef and dairy herds, respectively. In addition, in 20.8% (126) of beef and 18.4% (18) of dairy herds, cattle shared housing with the small ruminants. Indirect contact between species was reported in 39.9% (236) of beef and 52% (51) of dairy herds and no contact between them in 10.2% (61) and 25.5% (25) of beef and dairy herds, respectively.

#### Vaccination

Overall, 935 (22.8%) of all herds were reported as vaccinating at the time of investigation, with this being higher in dairy (29.7%) than beef herds (18.5%) (Table 6) and these proportions relatively stable between years. However, 412 of 934 herds for which responses were available reported that the vaccination regimen had been in place for <1 year, suggesting that it had been initiated after the BVD+ result(s) that triggered the investigation. Conversely, over 37% of herds with BVD+ births reported having a vaccination regimen in place for 3 or more years. Most of the vaccinated herds (63.6% of beef and 74.2% of dairy herds) reported using an inactivated vaccine (Table 7).

**Table 6:** Number (%) of herds applying BVD vaccination by herd type and year.

		**2016**	**2017**	**2018**	**2019**	**2020**	**Total**
Beef	Not Vacc	502	543	314	222	122	1,703
		81.1%	81.2%	83.7%	82.5%	77.2%	81.5%
	Vacc	117	126	61	47	36	387
		18.9%	18.8%	16.3%	17.5%	22.8%	18.5%
Dairy	Not Vacc	251	383	235	153	105	1,127
		66.1%	68.4%	74.8%	75.6%	72.4%	70.3%
	Vacc	129	177	79	52	40	477
		33.9%	31.6%	25.2%	25.4%	27.6%	29.7%
Dual	Not Vacc	80	127	65	42	25	339
		77.7%	84.1%	90.3 %	77.8%	83.3%	82.7%
	Vacc	23	24	7	12	5	71
		22.3%	15.9%	9.7 %	22.2%	16.7%	17.3%
Total	Not Vacc	833	1,053	614	417	252	3,169
		75.6%	76.3%	80.7%	79%	75.7%	77.2%
	Vacc	269	327	147	111	81	935
		24.4%	23.7%	19.3%	21%	24.3%	22.8%

**Table 7:** BVD vaccination by herd type, number of years vaccinating, and type of vaccine used.

**Years** **vaccinating**	**Beef**			**Dairy**			**Dual**			**Grand** **total**
	**Live**	**Inactivated**	**Total**	**Live**	**Inactivated**	**Total**	**Live**	**Inactivated**	**Total**	
<1	118	125	243	81	65	146	14	9	23	412
			62.8%			30.6%			32.9%	44.1%
1	11	37	48	15	28	43	5	0	5	96
			12.4%			9.0%			7.1%	10.3%
2	6	28	34	7	25	32	1	7	8	74
			8.8%			6.7%			11.4%	7.9%
3	4	13	17	3	27	30	1	7	8	55
			4.4%			6.3%			11.4%	5.9%
4	1	5	6	2	28	30				36
			1.6%			6.3%				3.8%
5	1	10	11	5	32	37		3	3	51
			2.8%			7.8%			4.3%	5.5%
>5		28	28	10	149	159		23	23	210
			7.2%			33.3%			32.9%	22.5%
Total	141	246	387	123	354	477	21	49	70	934
	36.4%	63.6%	100%	25.8%	74.2%	100%	30%	70%	100%	100%

### Source of Infection Analysis

One or more plausible sources were identified in 75% of all the investigations across the years (Table 8). Overall, plausible sources were found in 80.1% of beef and 68.2% of dairy herd investigations across the 5 years, with these levels being relatively consistent between years.

**Table 8:** Number (%) of cases reporting having found one or more plausible sources of infection by year and herd type.

	**2016**	**2017**	**2018**	**2019**	**2020**	**Total**
Beef	522	522	295	212	123	1,674
	84.3%	78.0%	78.7%	78.8%	77.8%	80.1%
Dairy	277	387	205	135	90	1,094
	72.9%	69.1%	65.3%	65.9%	62.1%	68.2%
Dual	85	107	57	36	25	310
	82.5%	70.9%	79.2%	66.7%	83.3%	75.6%
Total	884	1,016	557	383	238	3,078
	80.2%	73.6%	73.2%	72.5%	71.5%	75.0%

#### Within Herd Source

A summary of results for both within-herd sources and those external to the herd is presented in Table 9. The three most commonly identified plausible within-herd sources were Trojan dams, known PI animals retained within the herd and animals with false-negative results disclosed during the investigation. Overall, 20.9% (794) of investigations identified Trojan births as the plausible source of the outbreak, with the proportion of those being similar every year. A retained PI was reported as a plausible source of infection for 15.7% (644) of investigations over the 5 years, with the highest proportion being found for all three herd types in 2016 (16.1–21.4%). Animals with an apparent false-negative result detected during the investigation were identified as a source in 11.7% (481) of investigations overall, being reported more commonly in beef than in dairy herds. The presence of sheep was found as a plausible source of infection in 3.7% (150) of investigations over the years.

**Table 9:** Number (%) of herds in which plausible sources of infection either within or outside herds were identified overall and by year and herd type.

		**Beef**					**Dairy**					**Dual**					**Grand total**
		**2016**	**2017**	**2018**	**2019**	**2020**	**2016**	**2017**	**2018**	**2019**	**2020**	**2016**	**2017**	**2018**	**2019**	**2020**	
Within herd	Known PI retained in herd	129	128	52	46	27	61	82	28	16	12	22	21	6	9	5	644
		20.8%	19.1%	13.9%	17.1%	17.1%	16.1%	14.6%	8.9%	7.8%	8.2%	21.4%	13.9%	8.3%	16.7%	16.7%	15.7%
	Unid PI found during the investigation	26	14	7	5	4	11	6	8	1	1	5	3	0	2	2	95
		4.2%	2.1%	1.9%	1.9%	2.5%	2.9%	1.1%	2.%	0.5%	0.7%	4.9%	2.0%		3.7%	6.7%	2.3%
	Unid PI present during WOS that left the herd	51	21	6	12	2	23	20	9	6	3	14	5	3	0	1	176
		8.2%	3.1%	1.6%	4.5%	1.3%	6.1%	3.6%	2.9%	2.9%	2.1%	13.6%	3.3%	4.2%		3.3%	4.3%
	Introduced TI animal	10	12	12	5	5	2	6	4	1	2	1	0	1	2	0	63
		1.6%	1.8%	3.2%	1.9%	3.2%	0.53%	1.1%	1.3%	0.5%	1.4%	1.0%		1.4%	3.7%		1.5%
	Trojan birth	82	145	81	64	33	57	113	60	29	30	31	27	21	11	10	794
		19.4%	21.7%	21.6%	23.8%	20.9%	19.6%	20.2%	19.1%	14.2%	20.6%	36.1%	17.9%	29.2%	20.4%	33.3%	20.9%
	AFN disclosed during investigation	83	95	70	45	22	17	43	18	18	8	14	25	13	7	3	481
		13.4%	14.2%	18.7%	16.7%	13.9%	4.5%	7.7%	5.7%	8.8%	5.5%	13.6%	16.6%	18.1%	13.0%	10.0%	11.7%
	Presence of sheep	41	30	12	16	6	5	8	‘2	2	3	6	7	6	4	2	150
		6.6%	4.5%	3.2%	5.9%	3.8%	1.3%	1.4%	0.6%	1.0%	2.1%	5.8%	4.5%	8.3%	7.4%	6.7%	3.7%
Outside herd	Direct contact: boundary contact	224	181	96	89	44	142	174	92	65	49	22	45	20	11	8	1262
		36.2%	27.1%	25.6%	33.1%	27.9%	37.4%	31.1%	29.3%	31.7%	33.6%	21.4%	29.8%	27.8%	20.4%	26.7%	30.7%
	Direct contact: shared grazing	7	10	4	0	0	5	3	2	0	0	0	1	0	1	0	33
		1.1%	1.5%	1.1%			1.3%	0.5%	0.6%				0.7%		1.9%		0.8%
	Direct contact: returning cattle (TI)	59	38	20	12	9	21	20	18	7	4	8	13	5	3	0	237
		9.5%	5.7%	5.3%	4.5%	5.7%	5.5%	3.6%	5.7%	3.4%	2.7%	7.8%	8.6%	6.9%	5.6%		5.8%
	Indirect contact: herd owner	125	100	57	54	27	56	92	43	23	12	20	29	22	8	5	673
		20.2%	15.0%	15.2%	20.1%	17.1%	14.7%	16.4%	13.7%	11.2%	8.2%	19.4%	19.2%	30.6%	14.8%	16.7%	16.4%
	Indirect contact: other personnel	95	95	43	40	17	71	101	59	31	16	21	30	11	6	3	639
		15.4%	14.2%	11.5%	14.9%	10.8%	18.7%	18.0%	18.8%	15.1%	11.0%	20.4%	19.9%	15.3%	11.1%	10.0%	15.6%
	Indirect contact: small equipment	16	20	17	7	4	12	5	5	2	3	2	5	1	2	0	103
		2.6%	3.0%	4.5%	2.6%	2.5%	3.2%	0.9%	1.6%	1.0%	2.1%	2.3%	3.7%	1.4%	3.7%		2.5%
	Indirect contact: large facilities	54	53	23	19	6	33	31	16	6	3	6	16	11	3	1	281
		8.7%	7.9%	6.1%	7.1%	3.8%	8.7%	5.5%	5.1%	2.9%	2.1%	5.8%	10.6%	15.3%	5.6%	3.3%	6.9%
	Indirect contact: shared facilities	17	15	8	11	0	9	7	6	2	2	1	2	2	0	2	84
		2.8%	2.2%	2.1%	4.1%		2.4%	1.3%	1.9%	1.0%	1.4%	1.0%	1.3%	2.8%		6.7%	2.1%
Herds per year		619	669	375	269	158	380	560	314	205	146	103	151	72	54	30	4105

*PI, persistently infected; Unid, unidentified; WOS, window of susceptibility; AFN, apparent false negative; TI, transient infection*.

#### Source External to the Herd

The three most commonly identified plausible sources external to the herd were direct contact at boundaries, indirect contact via the herd owner and indirect contact via other personnel. Direct boundary contact with neighbouring herds was reported as a plausible source of infection external to the herd in 30.7% (1,262) of investigations (Table 9). Indirect contact both through the herd owner and other personnel were indicated as the probable source in 16.4 (673) and 15.6% (639) of investigations, respectively. The herd owner was more frequently identified as a plausible source in beef than in dairy herds, while the converse was found in relation to the role of other personnel.

### Test Results

#### 2016–2018: Testing of Animals With ‘Non-Negative’ Statuses

TASAH sampling carried out between 2016 and 2018 included all animals with “non-negative” statuses, confirmatory testing of virus-positive animals and the testing of the dams of PIs (DAMPI). Non-negative animals included in the list were those with the following statuses: EMPTY, INVALID, NONCOMP35, UNKNOWN (over 35 days of age), INTRODUCED35, DAMPI, and OFFPI; 7,066 animals with a DAMPI status were tested during these 3 years, of which 153 (2.2%) yielded a virus-positive result. An additional 10,415 animals were tested (4,687 in 2016, 3,296 in 2017 and 2,433 in 2018), comprising 5,249 that did not have a previous BVD result and 5,166 that did; 5.1% (529) returned a virus positive result and 0.1% (14) an inconclusive result. Of those that did have a previous result, 3,620 had “Negative,” 871 “Empty,” 611 “Positive,” 34 “Inconclusive,” and 31 “Invalid” results recorded. Overall, a total of 119 animals with apparent false-negative results were detected during this period (32 in 2016, 48 in 2017, and 39 in 2018).

#### 2019–2020 TASAH Sampling

A total of 7,849 animals were tested in 2019 and 14,527 in 2020. Of these, 56 were classified as AFN animals (26 in 2019 and 30 in 2020), including 10 DAMPI animals. A total of 1,978 DAMPI animals were tested during this period.

### Analysis of Biosecurity Recommendations

After completing the questionnaire and reviewing the biosecurity on farm, the PVP and the herd owner are required to agree on up to three measures to improve herd biosecurity. As the biosecurity recommendations are “free text,” these were reviewed and categorised in order to facilitate the analysis. Recommendations were categorised as relating to biosecurity; herd management and testing; management of BVD-positive animals; equipment; facilities; grazing; other species; personnel; and purchase, sale and vaccination policies.

The most widely reported recommendation over the 5 years related to the purchase of animals (24%, 2,731), including those in relation to the quarantine of animals prior to introduction (in terms of protocol, time period and facilities), followed by disinfection procedures, particularly those related to personnel, including the herd owner and visitors (20.3%, 2,302). Recommendations related to vaccination (19.7%, 2,242) and the risks of introduction of virus associated with contact with neighbouring cattle at pasture (17.5%, 1,992) were also commonly made.

## Discussion

Although the use of a systematic epidemiological investigation for some diseases of cattle may be common, for example, within bovine tuberculosis eradication programmes, it is not a tool that has been described in the literature in the context of a BVD disease eradication programme. However, it has some clear advantages that include the provision of a framework for the systematic collection of data from herds experiencing outbreaks and providing investigating PVPs with appropriate training and tools. Collection and analysis of data from these herds facilitates the monitoring of biosecurity breaches that are important for the spread of infection and helps to formulate biosecurity messages accordingly from a programme management point of view. Additionally, since biosecurity implementation is a key component of these programmes, the review process can identify and aim to correct any weaknesses to help the prevention of future outbreaks.

Recognition of the importance of biosecurity in the prevention and control of infectious diseases has increased substantially over the past few decades. It is now well-recognised that the prevention and control of diseases of animals through biosecurity practices can result in positive outcomes in terms of animal health and welfare. This was highlighted by the European Commission's Animal Health Strategy for the European Union (2007–2013), which focused on “prevention is better than cure” (29). Previous studies have suggested that the probability of introducing BVDV and BoHV-1 could be reduced by the implementation of biosecurity measures (30). However, limited information is available on the biosecurity practices within Irish farms. A previous study describing influences on biosecurity practices on Irish dairy farms found that >72% of farmers surveyed considered biosecurity to be important while 53% stated that a lack of information might prevent them from improving their biosecurity (28). In that study, farmers in the most dairy cattle-dense region were three times more likely to quarantine purchased stock than were their equivalents in regions where dairy production was less intense (*p* = 0.012). Younger farmers in general were over twice as likely as middle-aged farmers to implement biosecurity guidelines (*p* = 0.026). The importance of biosecurity in disease control in Ireland has been highlighted in the National Farmed Animal Biosecurity Strategy (2021–2024) (31), launched by the DAFM in early 2021, which reinforces the shift in emphasis toward disease prevention and a focus into raising the standard of biosecurity on all Irish livestock farms.

In the absence of an eradication programme, the risk of BVD infection from animal introductions may originate, in decreasing order of likelihood, from BVD+ animals, Trojan dams and transiently infected animals (1, 15, 22). Within the Irish BVD programme, all cattle born after January 1, 2013 must have a negative virus test result to move and the majority of older females will have produced at least one negative calf and therefore have an indirect negative status (INDINEG). Since May 2020, the small number of animals born prior to January 1, 2013 without a known status must also be tested and have a negative BVD test result for trade purposes. Therefore, the risk of introducing a PI animal is very low, although it may still happen if the animal has a false-negative test. In the context of the Irish programme, an AFN result occurs when an animal returns a positive or inconclusive result having recorded a previous negative result. At the end of 2020, a total of only 260 animals born between January 1, 2013 and December 31, 2020 had been identified as AFNs (14). However, the disclosure of an AFN was recorded as a plausible source of infection in 481 investigations. This discrepancy could be due to a lack of understanding or a suspicion that an AFN had been present in the herd during the WOS without one having actually been identified. The identification of AFN animals is key to remove all sources of infection in the investigated herds and thus achieve the objectives of the investigation.

Trojan births were reported in 20.9% of investigations as a likely source of infection. The role of Trojans in the epidemiology of BVD in Ireland has been highlighted before. A previous study that reviewed Trojan births in Ireland during 2013–15 found that over those years, the percentage of BVD+ birth events attributable to Trojan dams increased each year, being 7.1% in 2013, 9.2% in 2014, and 10.6% in 2015 (9); and a further study found the purchase of cattle including potential Trojan cattle as one of the risk factors associated with the loss of Negative Herd Status in 2017 (20). The relative importance of Trojans has been considered to become more significant as a programme progresses (32), which fits with the higher proportion of infections due to Trojan births in the most recent years. Where a Trojan dam was identified as a plausible cause of a BVD+ birth, the source herd where the dam was located during the WOS was identified to determine if a recognised source of infection was present, triggering the required investigations in the source herd. Where infection was not identified, suggesting either a breach of biosecurity or the presence of an unidentified source of infection, a separate investigation was assigned to the source herd.

Transiently infected animals are considered a much lower risk than PI animals but cannot be excluded (15). Following transient infection, virus is typically shed at low levels and for a short period (up to 14 days) (6, 25, 33). A key element of biosecurity to minimise the risk of introduction of infection through TI animals is the implementation of quarantine for at least 4 weeks. Only 17.5% of herds had a quarantine policy for introduced animals and of those, 13% did not apply it to the animals introduced to the herd during the WOS. The general lack of application of correct quarantine procedures was reflected on the biosecurity recommendations, where the implementation of good biosecurity practises around purchase of cattle generally (avoid purchasing of pregnant animals, introduce a closed herd policy, etc) and of quarantine (for at least 4 weeks, in a separate building or paddock etc) featured widely. Furthermore, some PVPs suggested shorter quarantine periods, indicating lack of consensus or best practise on this measure. This general lack of understanding of the application of quarantine measures has also been highlighted in previous studies (28, 34).

Prompt identification and removal of BVD+ calves are critical to ensuring that optimum progress is made in the BVD eradication programme. Previous Irish studies have shown that retention of BVD+ calves into the breeding season increases the likelihood of further PI births (17). Of the investigated herds, 15.7% reported a retained PI animal as the plausible source of infection, with this being more frequent in beef (18.3%) than dairy herds (12.4%). Retained animals were one of the key challenges in the early years of the programme (14, 35). The introduction of a series of measures has largely resolved this issue, including graduated financial supports for their removal, movement restrictions, ongoing programme communications and the input of PVPs.

BVD virus can infect other ruminant species (sheep, llamas, alpacas) and pigs (24, 36). Transmission between small ruminants and cattle, both ways, has been demonstrated (37, 38). BVDV has been detected in sheep in Ireland, but at a lower flock and animal level prevalence than that seen in cattle, suggesting that the main direction of transfer is from cattle to sheep rather than sheep to cattle (39, 40). The low proportion of investigations highlighting the presence of small ruminants as a plausible source of infection agrees with findings from previous studies where the presence of sheep was not associated with the herd having virus-positive results (20, 22).

The plausible source of infection from outside of the herd indicated most often was direct boundary contact with neighbouring herds (30.7% of investigations). Contiguous spread has been identified as a plausible transmission pathway in the BVD eradication programme in Ireland, due to the highly fragmented nature of land holdings on many Irish farms (16). The density of BVD infection within 10 km of the herd emerged as a significant factor associated with the loss of the Negative Herd Status in 2017 for herds in the Irish programme (20). While not indicating that herds were contiguous, the density of infection provides an indication of the probability of neighbouring herds being infected and, therefore, of the risk that contact with neighbouring cattle may entail. A recent meta-analysis of risk factors associated with BVD also found significant higher odds for herds that share pasture or have direct contact with cattle of other herds at pasture (11).

Indirect contact through the herd owner or personnel was reported as a plausible source in 16.4% and 15.6% of investigations, with differences between beef and dairy herds. Beef herds reported a higher proportion of sources involving the herd owner (17.4%) than personnel (13.9%), reflecting the part-time nature of many beef enterprises. Conversely, dairy herds reported a higher proportion of sources to personnel (17.3%) than to the herdowner (14.1%), reflecting a more business-like structure, where farm staff and a wide range of professional service providers are more common. Even though the role of indirect transmission is more difficult to demonstrate and quantify, different studies have attempted to clarify its impact on BVD transmission (7, 27). In the current study, among people coming into contact with cattle, PVPs were the visitors reported as having visited the farms more often. Although veterinarians have been previously linked with a higher probability of introducing BVDV (30), frequency of visiting alone is not enough to infer transmission as other biosecurity measures such us cleansing and disinfection, changing of clothing, etc., will contribute to the control of infection, with these also being assessed in each investigation and taken into consideration when determining their plausibility as a source of infection. A study into the BVDV-2 outbreak in Germany in 2012 found that the virus was mainly transmitted by person contacts, and also by cattle trade and vehicles (8).

The proportion of vaccinating herds was higher among dairy (29.7%) than beef herds (18.5%), with these levels not changing significantly throughout the years considered in this paper. 36.4% of vaccinating beef herds and 25.8% of dairy used live vaccine. This is in contrast with details of annual vaccine sales, obtained through a market research company^3^, that showed that total doses sold in Ireland have experienced a 32.2% decrease since 2016/17, with sales of inactivated vaccines predominating (14). However, when the length of time in years that the vaccination regimen has been in place is analysed, between 42.3 and 49.4% of investigated herds through the years indicated they had been vaccinating for <1 year, which is considered to reflect the initiation of a vaccination regimen on the basis of the PVP's recommendation following the disclosure of the positive result. Veterinary recommendation of live vaccine is also considered to be the basis for the much higher proportion of usage in investigated herds than generally. The finding that up to 25% of investigated herds were vaccinating for over 5 years is a concern. The birth of PI calves in these herds could reflect sub-optimal storage, application or efficacy of vaccines, as well as the birth of PI calves to Trojan dams.

Compliance with completion of investigations was generally high from 2017 onwards, when they became compulsory. Although a lower proportion of completed investigations were recorded for 2020, at the time of writing, investigations from herds that disclosed positive results at the end of the year were still pending.

One of the limitations of the herd investigation framework is the possibility of recall error in the herd owners' responses. Therefore, PVPs were encouraged to back up, as far as possible, any findings with the data available in the programme's database. Nonetheless, the findings in terms of plausible sources in these herds are validated by other studies which also highlight the roles of introduced animals, Trojans, local PI density and neighbouring herds (16, 20). Another limitation for applying the findings more widely is that they are gathered specifically in the context of BVD, from herds with positive or inconclusive virus result(s). Nonetheless, this review highlights several areas of weakness in biosecurity in these farms that the authors consider to require attention more generally in terms of minimising the risk of introduction of infectious diseases. These include the frequent contact with neighbouring cattle on farm boundaries, including break-ins/outs and herd owner, personnel and visitors' lack of personal biosecurity in terms of disinfection and use of separate clothing before coming into contact with the herd's animals. Also, deficiencies in the understanding and implementation of quarantine measures were noted. While infrequent, general low levels of cleaning and disinfection of borrowed equipment and shared facilities are also important to note. These findings agree with previous studies that have revealed low implementation of biosecurity measures at farm level in other countries (41, 42).

The herd investigation framework described here provides a structured approach to investigating BVD breakdowns. Although it will not always be possible to identify plausible sources of infection, the structured approach to investigating breakdowns identifies the window of susceptibility for each dam that produced a BVD+ calf and seeks to identify possible direct or indirect means by which exposure could have happened during this time. Even where the source is not definitively identified, working through this process will identify weaknesses in biosecurity and allow recommendations to correct these to be made. While this herd investigation tool is focused on BVD, it provides an overview of some of the biosecurity shortcomings of the Irish industry. In addition, the implementation of the biosecurity recommendations will typically produce wider benefits in relation to improving or maintaining herd health.

## Data Availability Statement

The raw data supporting the conclusions of this article will be made available by the authors, without undue reservation.

## Author Contributions

MG-G and DG prepared the manuscript. J-ML, EL, and PO'S reviewed it. EL, J-ML, and PO'S are members of the BVD TWG and IG. All authors contributed to the article and approved the submitted version.

## Conflict of Interest

The authors declare that the research was conducted in the absence of any commercial or financial relationships that could be construed as a potential conflict of interest.

## Publisher's Note

All claims expressed in this article are solely those of the authors and do not necessarily represent those of their affiliated organizations, or those of the publisher, the editors and the reviewers. Any product that may be evaluated in this article, or claim that may be made by its manufacturer, is not guaranteed or endorsed by the publisher.

## Footnotes

^1^ www.icbf.com

^2^ www.animalhealthireland.ie

^3^ www.kynetec.com
